# *Lentinula edodes* as a Source of Bioactive Compounds with Therapeutical Potential in Intestinal Inflammation and Colorectal Cancer

**DOI:** 10.3390/ijms26073320

**Published:** 2025-04-02

**Authors:** Mikołaj Bugajewski, Norbert Angerhoefer, Leszek Pączek, Beata Kaleta

**Affiliations:** 1 Students Scientific Society, Department of Clinical Immunology, Medical University of Warsaw, Nowogrodzka 59, 02-006 Warsaw, Poland; mikolajbugajewski@gmail.com (M.B.); norbertang8@gmail.com (N.A.); 2 Department of Clinical Immunology, Medical University of Warsaw, Nowogrodzka 59, 02-006 Warsaw, Poland; leszek.paczek@gmail.com; 3 Institute of Biochemistry and Biophysics, Polish Academy of Sciences, Pawinskiego 5a, 02-106 Warsaw, Poland

**Keywords:** colorectal cancer, Crohn’s disease, functional food, gut, inflammatory bowel disease, *Lentinula edodes*, ulcerative colitis

## Abstract

Inflammatory bowel disease (IBD), including Crohn’s disease (CD) and ulcerative colitis (UC), is a rising global health issue. Chronic intestinal inflammation is an important risk factor for colorectal cancer (CRC). Despite significant progress in IBD and CRC treatment, numerous patients remain resistant to standard pharmacotherapy or experience severe side effects that prevent them from continuing treatment. There is evidence suggesting that bioactive substances in *Lentinula edodes* have immunomodulatory and anticancer properties. This fungus is currently classified as a functional food, considering its beneficial effects on human health and special nutritional value. Studies conducted in vitro and in animal models demonstrated that *L. edodes* bioactive compounds, in particular glucans, have anti-inflammatory and antioxidant effects, induce apoptosis of cancer cells, reduce tumor angiogenesis, restore gut microbiome heterogeneity and improve gut barrier dysfunction. Moreover, clinical trials confirmed that these compounds combined with standard chemotherapy have a significant effect in improving the prognosis of CRC patients. In addition, *L. edodes* glucans increase microbial diversity and enhance interferon (IFN)-γ production by immune cells. Future studies must be focused on understanding the pathways and mechanisms associated with the observed effects. Moreover, both randomized trials and long-term follow-up studies are needed to confirm their effectiveness in the treatment of IBD and CRC.

## 1. Introduction

The gastrointestinal tract (GIT) is a complex organ system that, in addition to digestion, absorption and excretion, plays a significant role in the regulation of immunologic and endocrine function [1]. The GIT contains four histological layers composed of various tissues with different functions: the mucosa, submucosa, muscularis propria and serosa [2]. The mucosa is the innermost layer, composed of three layers: (1) epithelium cells, responsible for digestion, absorption and secretion; (2) lamina propria, which provides nutrients to the epithelium and contains immune cells, including T and B cells, neutrophils and macrophages; and (3) the muscularis mucosae, which is a layer of smooth muscle responsible for local movement of the mucosa located outside the lamina propria [3]. The submucosa is a layer of loose connective tissue located under the muscularis mucosa, containing blood and lymphatic vessels, nerves, as well as submucosal glands [3]. The third layer is muscularis propria. It is composed of two layers of smooth muscle cells and is responsible for the peristaltic movements. The outermost layer of the GIT, called the serosa, contains several layers of connective tissue, as well as blood, lymphatic vessels and nerves [2,3].

The intestinal epithelium is the first line of defense in the gut [1]. Numerous intestinal barrier damage risk factors have been identified and described. The most important ones are genetic factors, microbial dysbiosis, environmental factors and allergens [2,4]. It has been demonstrated that the loss of epithelial barrier integrity, referred to as “leaky gut syndrome”, contributes to numerous disorders, including neuropsychiatric diseases (Parkinson’s disease, Alzheimer’s disease), diabetes mellitus types 1 and 2, psoriasis, multiple sclerosis, rheumatoid arthritis, inflammatory bowel disease (IBD) and colorectal cancer (CRC) [4].

Inflammatory bowel disease (IBD) is a group of idiopathic, chronic inflammatory conditions affecting the integrity of the GIT. IBD is divided into two major types: Crohn’s disease (CD) and ulcerative colitis (UC) [5]. The incidence of both CD and UC has risen over the last decades to become global emergence diseases [6]. Although the clinical phenotype of both disorders is similar, CD and UC can be distinguished by different localization, immunological and histological parameters. IBD commonly develops between the ages of 15 and 35 years; nevertheless, it can build up at any time [5]. Usually, CD affects the mouth, intestines and anus; however, lesions can occur in any part of the GI tract. UC occurs specifically in the mucosal layer of the colon and rectum [7]. CD symptoms are similar to UC and include diarrhea, rectal bleeding (less common in CD), abdominal pain, fatigue and weight loss [8]. Both CD and UC result from an inappropriate inflammatory response to commensal bacteria, which results from exposure of a genetically susceptible person to environmental factors, such as saturated fatty acids, processed meats, tobacco, alcohol, antibiotics, nonsteroidal anti-inflammatory drugs, anti-contraceptives and many others [9,10,11,12,13]. It has been confirmed that dysregulation of innate and adaptive immune responses in the intestinal mucosa plays a key role in IBD etiology [14]. Macrophages, dendritic cells (DCs), neutrophils, natural killer (NK) cells and innate lymphoid cells (ILCs) are the first line of defense against pathogens. They are responsible for the synthesis of pro-inflammatory cytokines, chemokines and antimicrobial agents, phagocytosis, presentation of antigens to T cells and their activation [15,16,17]. Subsequent to activation, intestinal naïve T cells differentiate into a variety of T helper (Th) cell subsets characterized by different functions and cytokines production, T regulatory (Treg) and T memory (Tm) cells [18]. It is believed that the CD is associated with Th1/Th17 immune response, while the UC is mediated by Th2 cells [19]. In the IBD course, activated Th1 cells secrete pro-inflammatory interferon (IFN-γ), tumor necrosis factor (TNF-α) and interleukin (IL-2). Moreover, Th1 stimulation results in the activation of signal transducer and activator of transcription-1 (STAT1) and upregulation of the T-box transcription factor TBX21 (T-BET), which in turn promotes the recruitment of macrophages, NK cells and cytotoxic T cells [16,18]. Moreover, Th17 cells produce pro-inflammatory cytokines such as IL-17A, IL-21 and IL-22 and play a major role in IBD pathogenesis [19]. In contrast, Th2 cells synthesize cytokines (IL-4, IL-5 and IL-13), which inhibit Th1 cells and upregulate the activation of macrophages. Treg cells play a significant role in IBD. These cells express the transcription factor forkhead box P3 (FOXP3), cytotoxic T-lymphocyte antigen 4 (CTLA-4) and produce anti-inflammatory cytokines, including IL-10 and transforming growth factor (TGF-β), which suppress immune cells response in guts [5,14]. Tissue-resident memory T cells have been likewise suggested to play an important role in IBD pathogenesis. These cells also produce pro-inflammatory cytokines and chemokines, which results in the recruitment of DCs and NK cells [18].

**Figure 1:**
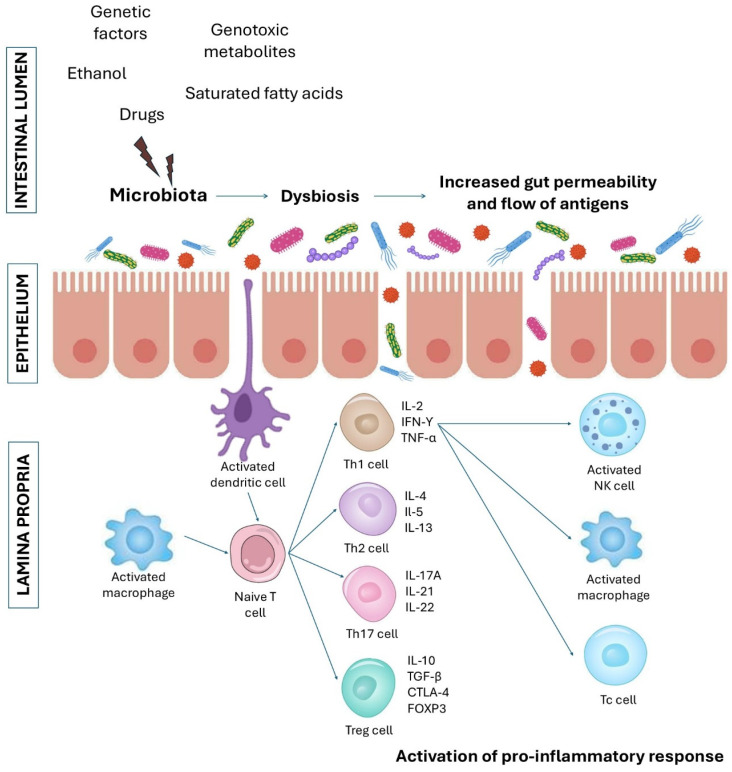
Schematic representation of the pathogenic mechanism of chronic gut inflammation associated with increased intestinal permeability. Environmental factors and genetic predispositions cause microbial dysbiosis, loss of permeability, increase in the flow of antigens from the intestinal lumen to the lamina propria and activation of pro-inflammatory response, which is a main cause of the development of inflammatory bowel diseases and colorectal cancer. NK, natural killer; Tc, cytotoxic T cell; Th, T helper cell; Treg, regulatory T cell.

Patients with IBD are at increased risk of developing colorectal cancer (CRC) [20]. The exact mechanism behind this association is not fully understood; however, it was suggested that chronic intestinal inflammation is the risk factor for the development of dysplasia [21]. Moreover, in IBD patients, elevated nitric oxide synthase activity, as well as reactive oxygen species production, have been found [22]. It was also confirmed that Treg lymphocytes induce epithelial apoptosis [23]. Recently, several studies have reported that some metabolites from the diet, as well as dysbiotic gut microbiota-derived metabolites, induce DNA damage and dysplasia [23,24] (Figure 1).

Taking the above into consideration, improving intestinal barrier function and maintaining a more diverse and balanced gut microbiome is of great significance for the prevention and treatment of chronic inflammatory conditions of the intestine and CRC. The standard IBD treatment is a combination of self-care and pharmacotherapy [25,26]. In mild and moderate phases of the disease, amino-salicylates (mesalamine, sulfasalazine, balsalazide) are the first-line therapy. In patients with more severe conditions, corticosteroids are used temporarily. However, their long-term use is associated with an increased risk of adverse drug reactions [26,27]. Therefore, in patients for whom these drugs are ineffective, or if their use is not possible, immunomodulators are used. Immunosuppressants, such as azathioprine and 6-mercaptopurine are characterized by high effectiveness and safety [27]. IBD pharmacotherapy has been revolutionized by the use of biological drugs, including monoclonal antibodies against TNF-α (infliximab, adalimumab, golimumab), integrins (vedolizumab and natalizumab), IL-12 and IL-23 (ustekinumab). Novel therapeutic options, including small molecules, have been approved to treat moderate and severe IBD. These include Janus kinases (JAK) and tyrosine kinase 2 (TYK2) inhibitors, modulators of S1P receptor, phosphodiesterase 4 (PDE4) inhibitors, as well as toll-like receptor 9 (TLR9) agonists [25,26,27,28]. Unfortunately, numerous IBD patients do not respond to available therapies or experience secondary loss of response. Therefore, there is a growing need to find new therapeutic options to control IBD.

Despite significant research progress in CRC therapy, this malignancy is the second leading cause of cancer-related deaths worldwide [29]. Currently, CRC therapy includes surgical resection, single-agent chemotherapy (mainly with fluoropyrimidines) or multiple-agent chemotherapy (mainly with oxaliplatin, irinotecan and capecitabine) and radiotherapy [30]. However, chemotherapy is associated with certain limitations; therefore, new strategies have been proposed, including target therapy with anti-vascular endothelial growth factor/vascular endothelial growth factor receptor (VEGF/VEGFR) antibodies (bevacizumab, ziv-aflibercept, regorafenib, ramucirumab, fruquintinib), anti-epidermal growth factor receptor (EGFR) antibodies (cetuximab, panitumumab), anti-B-Raf proto-oncogene serine/threonine kinase (BRAF) inhibitors, anti-MEK inhibitors (vemurafenib, dabrafenib, encorafenib, trametinib, binimetinib), anti-HER2 antibodies (trastuzumab, pertuzumab, lapatinib, tucatinib), anti-KRAS antibodies (cetuximab, panitumumab) and RET inhibitors (selpercatinib) [30,31,32]. However, some CRC patients are resistant to these drugs or experience severe side effects that prevent them from continuing treatment; therefore, there is a growing need to find effective, available and affordable options, including natural products, functional foods and nutraceuticals.

Over the past 40 years, various natural products have been approved for the treatment of numerous chronic disorders, including cancer and inflammatory diseases. Medicinal mushrooms have been used for hundreds of years to promote health, and many mushroom-derived bioactive compounds have been applied as adjuvants for standard therapies in Asian countries [33,34]. One of them is *Lentinula edodes* (shiitake mushroom). It is one of the most commonly cultivated edible mushrooms in the world. It is currently classified as a functional food considering its beneficial effects on human health and special nutritional value [33]. Numerous in vitro and in vivo investigations revealed that *L. edodes* fruiting bodies and mycelium are a source of various nutrients and bioactive substances with multiple beneficial health effects. The most active components in *L. edodes* are polysaccharides, especially α- and β-glucans [34]. The biological activity of polysaccharides depends on the methods of their extraction and *L. edodes* cultivation since it affects monosaccharide composition, molecular weight (Mw), branching degrees and helical conformation. Recent studies have demonstrated that polysaccharides with higher Mw exhibit elevated biological activity, which is associated with their ability to form triple-helix structures [33].

The molecular mechanisms through which fungal glucans exhibit biological effects are not fully elucidated; however, they may result from their complex interaction between the immune system (via receptors expressed on immune cells, including dectin-1, complement receptor type 3 [CR3], lactosyceramide [LaCer], scavenger, toll-like receptors [TLRs] and CD28 receptors) and various metabolic and epigenetic pathways [34]. *L. edodes* polysaccharides are referred to as biological response modifiers (BRMs) and, therefore, are considered promising agents for use in the treatment of inflammatory diseases and cancer.

This review, for the first time, presents current research on the role of various active compounds from *L. edodes* on GIT and summarizes the current state-of-the-art and future perspectives for their use in IBD and CRC therapy. 

## 2. *Lentinula edodes*-Derived Bioactive Compounds

**Figure 2:**
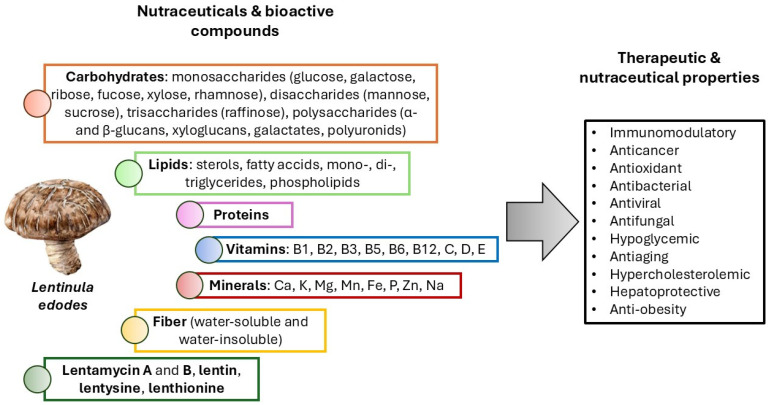
The main bioactive compounds and nutrients from *Lentinula edodes* and its therapeutic properties.

As mentioned above, *L. edodes* is an important source of numerous nutrients and various bioactive compounds (Figure 2). *L. edodes* is a good source of carbohydrates (38–66 g/100 g, including mono-, di-, tri- and polysaccharides), proteins (17–27 g/100 g), lipids (1.3–3 g/100 g), water-soluble and water-insoluble dietary fiber (2.5–30 g/100 g), vitamins, including vitamin B1 (0.3–0.07 mg/100 g), B2 (0.5–1.0 mg/100 g), B3 (14 mg/100 g), B5 (22 mg/100 g), B6 (0.95 mg/100 g), B12 (4.5–5.5 µg/100 g), C (2.1–3.5 mg/100 g), D (4 µg/100 g) and E (10 µg/100 g) and minerals: calcium (11 mg/100 g), potassium (1.5 g/100 g), magnesium (132 mg/100 g), manganese (1.2 mg/100 g), iron (1.7 mg/100 g), phosphorus (294 mg/100 g), zinc (7.7 mg/100 g) and sodium (13 mg/100 g) [33,34,35,36]. Since the dawn of the 21st century, there has been an increase in the studies on *L. edodes* cultivation and its application as a functional food. Recently, bioactive compounds, in particular polysaccharides isolated from *L. edodes* fruiting bodies or mycelium, have been shown to serve as important immunomodulators and potential antitumor agents [35,36,37,38]. It is necessary to emphasize that not only polysaccharides show biological activity. Vitamins (B and D) are able to regulate immune cell function, including T and B cells, natural killer (NK) cells, monocytes and macrophages, as well ad dendritic cells. Moreover, it has been found that Mg and Zn present in *L. edodes* are essential for the innate and adaptive immune response [39]. It was also indicated that *L. edodes* amino acids play a significant role in T, B, NK cells and macrophage activation, as well as cytokines, antibodies and nitric oxide (NO) production [33,39]. Numerous studies revealed that some secondary metabolites present in *L. edodes* (such as lentamycin A and B, lentithionine, lentysine) have direct and indirect antiviral, antimicrobial and antifungal potential [40]. Additionally, hypocholesterolemic and hypoglycemic actions of lentinacin (an S-adenosyl-L-homocysteine hydrolase inhibitor) have been reported [33,39].

## 3. The Effects of *L. edodes*-Derived Bioactive Compounds on Intestinal Barrier and Gastrointestinal Diseases

As mentioned above, currently, there are numerous therapeutic agents for the treatment of IBD and CRC; however, many patients fail to respond to treatment or experience serious side effects. Therefore, there is growing interest in developing effective, non-toxic and inexpensive drugs of natural origin.

Several studies conducted in vitro on cell lines, in animal models as well as in humans, demonstrated that natural bioactive substances from foods or plants improve the pathological symptoms of IBD, regulate intestinal microbiota, protect intestinal barrier integrity, downregulate pro-inflammatory responses and reduce oxidative stress [41].

### 3.1. Studies Performed on Cell Culture and Animal Models

It was confirmed that oxidative stress plays a significant role in the pathogenesis of CRC since it increases reactive oxygen species production, lipids peroxidation and DNA damage [22,23]. Oxidative stress induces nuclear factors crucial for antioxidant defense and carcinogenesis prevention, including nuclear factor-erythroid 2 (NF-E2), p45-related factor-2 (Nrf2) and nuclear factor-kappa B (NF-κB). Therefore, antioxidants, which are abundant in many functional foods, can contribute to the reduction in oxidative stress and inflammation. Thus, Takahashi et al. [42] analyzed the antioxidant potential of Active Hexose Correlated Compound (AHCC™), an enzyme-fermented water extract of the mycelia of *L. edodes* in human colon cancer cell lines (HCT116 and DLD-1) and in Apc mutant Min mice. AHCC is a mixture of polysaccharides, amino acids, lipids and minerals. Polysaccharides are the major components of AHCC, consisting of about 74% of the extract. About 20% of this fraction is composed of the α-1,4-glucans and their acetylated forms with Mw ~ 5 kDa [43]. The group demonstrated that AHCC dose-dependently increased Nrf2 and NF-κB activity in both cell lines. Moreover, it dose-dependently upregulated mRNA expression of heme oxygenase-1 (HO-1) and NAD(P)H:quinone oxidoreductase (NQO1), nevertheless reduced IL-6 mRNA expression. AHCC administered in the diet of Apc mutant Min mice decreased the number of intestinal polyps and lowered IL-6 production. It was suggested that the observed activity of the AHCC mixture was associated with the presence of α-1,4-glucans, which are considered BRMs.

In another study, Doursout and colleagues [44] analyzed the ability of AHCC to restore immune function in rats with LPS-induced inflammation in the gut and impaired nitric oxide signaling pathway. AHCC significantly reduced LPS-induced mean arterial pressure and pro-inflammatory cytokines (IL-1, IL-6, TNF-α) concentration. Additionally, AHCC decreased myeloperoxidase (MPO) levels in the gut, downregulated NO production and reduced lymphocyte infiltration and gut edema.

You et al. [45] analyzed the impact of lentinan in combination with probiotics on immunosuppression and oxidative stress responses in a dextran sulfate sodium (DSS)-induced colitis mice model. Lentinan is a highly purified (1-3;1-6)-β-D-glucan extracted from *L. edodes* fruiting bodies (hot water extraction) with Mw ~ 500 kDa triple-helical conformation. C57BL/6 mice were intraperitoneally injected with lentinan (5 mg/kg, 10 mg/kg or 20 mg/kg) once a day for 7 days. Lentinan, in a dose-dependent manner, reduced weight loss, disease activity index as well as colon shortening. Moreover, lentinan increased glutathione activity downregulated malondialdehyde and TNF-α, IL-6 and IL-1β levels in the colon. In addition, lentinan and probiotics treatment increased cell proliferation, reduced apoptosis and blocked the NF-κB pathway.

As mentioned above, lentinan is the most studied active compound isolated from *L. edodes*. Numerous studies have demonstrated its immunomodulatory and anticancer activity. However, only a few studies suggested its effects against IBD or CRC.

Nishitani et al. [46] analyzed the anti-inflammatory activity of lentinan in in vitro co-cultures of Caco-2 cells and LPS-stimulated macrophage RAW264.7 cells, as well as in dextran sulfate sodium (DSS)-induced colitis mice model. In Caco-2 cells, lentinan downregulated IL-8 mRNA expression and NF-κB activation, while it had no effect on TNF-α production in RAW264.7 cells. Moreover, it decreased the number of TNF receptor 1 (TNFR1) on the surface of Caco-2 cells. In addition, it was revealed that oral administration of lentinan (100 µg) reduced weight loss, prevented shortening of the colon length and downregulated IFN-γ, IL-1β and TNFR1 mRNA intestinal expression.

In the later study, the group focused on explaining the mechanism responsible for the observed effects and analyzed interactions between lentinan and dectin-1, the well-known β-glucan receptor [47]. Colitis was induced by DSS in Dectin-1-deficient mice (dectin-1 KO). A total of 100 µg of lentinan was administered daily. In wild-type (WT) mice similar effects were observed as in the previous study [41]. However, in dectin-1 KO mice, lentinan did not reduce colitis symptoms. Moreover, it was confirmed that lentinan reduced TNFR1 expression on Caco-2 cell membranes through dectin-1 ligation.

Minato et al. [48] compared the effect of oral and rectal lentinan administration on DSS-induced colitis and examined the migration of CD4^+^ T cells from the ileum to the colon in Kikume Green-Red (KikGR) mice. It was demonstrated that orally administered lentinan induced the recruitment of Th and Treg cells from an ileum into a colon. Moreover, oral lentinan administration non-significantly downregulated IFN-γ, IL-1β, IL-4, IL-10 and TNF-α expression. In contrast, rectal administration did not change IL-6 and IL-10 production.

The effects of lentinan on HT-29 human colon cancer cells and HT-29 tumor-bearing athymic nude mice were also investigated by Wang et al. [49]. It was found that lentinan induced HT-29 cell apoptosis in a dose-dependent manner both in vitro and in mouse models, which was associated with caspase-3 activation. Moreover, it was revealed that lentinan activated caspase-8 and -9. Caspase-8 mediates extrinsic, and caspase-9 initiates the intrinsic pathway of apoptosis [50]. Lentinan also upregulated cytochrome c and the Bax/Bcl-2 ratio, reduced NF-κB activation, ROS levels and TNF-α production in vitro and in vivo. In the subsequent analyses, this group assessed the antitumor effect of lentinan in immunodeficient NOD/severe combined immunodeficiency (SCID) mice [50]. It was found that tumor volumes and weights of mice treated with lentinan (1 and 5 mg/kg) were significantly lower than in the control group. Moreover, autophagy-related proteins (LC3-II and Beclin-1), as well as autophagic flux, were upregulated in HT-29 cells. Additionally, lentinan activated Ca^2+^-induced apoptosis by activating inositol 1,4,5-trisphosphate receptor (IP3R) in vitro and in mice and upregulated endoplasmic reticulum stress (ERS), which can also induce autophagy and apoptosis [51].

It was suggested that lentinan can improve gut microbiota dysbiosis. Therefore, Ji et al. [52] investigated its therapeutic effects on broad-spectrum antibiotics-induced gut microbial dysbiosis in C57BL/6 J mice. Ampicillin, neomycin sulfate, metronidazole, vancomycin and lentinan (200 mg/kg/day) were administered orally for 14 consecutive days. It was demonstrated that lentinan administration reversed the dysbiosis induced by broad-spectrum antibiotics and increased the abundance of beneficial microbiota (S24-7, *Lactobacillus*, *Oscillospira*, *Ruminococcus* and *Allobaculum*). In addition, lentinan increased the levels of propionic and butyric acids, essential for microbiome homeostasis. Moreover, it was found that lentinan improved colon tissue morphology, increased the expression of tight-junction proteins such as ZO-1 and Occludin in mice and downregulated pro-inflammatory cytokines production via the NF-κB signaling pathway. It was proposed that lentinan can be used as a prebiotic.

Similarly, Liu et al. [53] found that lentinan effectively restored the gut microbiome heterogeneity in a mouse model of IBD and colitis-associated cancer (CAC). Moreover, lentinan decreased the disease activity index, and colon tissue damage reduced the number of tumors, colon cancer markers, immune cell infiltration and expression of pro-inflammatory cytokines such as IL-13.

Angiogenesis plays a crucial role in tumor growth and metastasis in CRC; therefore, antiangiogenic agents are currently widely used against CRC [54]. However, due to drug resistance and significant toxicities, the development of new antiangiogenic agents is crucial. Therefore, the influence of lentinan on tumor vessels was analyzed in mice inoculated with CT26 colorectal cells [55]. Lentinan treatment (1.0 mg/kg) significantly reduced tumor angiogenesis and tumor growth. Interestingly, lentinan in a dose of 4.0 mg/kg showed no such effect. Later, it was confirmed that the drug inhibited tumor angiogenesis via upregulation of IFN-γ production by enhancing tumor infiltration of IFN-γ-expressing cells (CD4^+^, CD8^+^ T cells, NK cells, neutrophils and monocytes).

Selenium (Se) is an essential trace element that plays a significant role in immunomodulation and redox regulation. Some studies suggested that low Se levels are associated with an increased risk of cancer, including CRC [56]. Se nanoparticles (SeNPs) have been characterized as having great bioavailability and bioactivity with low toxicity [57]. It has been demonstrated that natural polysaccharides can be used for SeNP stabilization with additional bioactive properties [58]. Therefore, Gao and colleagues [59] synthesized lentinan-functionalized SeNPs (named LNT-SeNPs) and analyzed their cytotoxic effects against colon cancer cell lines (HCT-116, HT-29, Caco-2, SW620 and CT26). SeNPs were prepared by mixing lentinan solution (0–8 mL, 2.5 mg/mL) with sodium selenite (Na_2_SeO_3_; 1 mL, 50 mM) under magnetic stirring. LNT-SeNPs were monodisperse spherical particles in the solution with an average diameter of 59.2–386.8 nm (depending on lentinan concentration). LNT-SeNPs (at the concentration range of 2–64 μM) inhibited the proliferation of all colon cancer cells. The strongest effect was observed for the HCT-116 cells. In addition, LNT-SeNPs increased apoptosis of HCT-116 cells and induced G0/G1 phase arrest in these cells. The authors concluded that LNT-SeNPs can be applied in CRC therapy.

In another similar study, Liu et al. [60] incubated CT26 mouse colon carcinoma cells with LNT-SeNPs (at the concentration range of 1–64 μM) for 72 h and demonstrated that LNT-SeNPs decreased CT26 cells viability and induced excessive ROS production in a dose-dependent manner. Moreover, similarly to Gao et al., Liu et al. demonstrated that LNT-SeNPs induced apoptosis and G0/G1 phase arrest. LNT-SeNPs, in combination with TNF-α, significantly inhibited tumor growth in CT26 tumor-bearing mice after 2 weeks of treatment.

Lentinan-based oral NPs loaded with budesonide (drug used in IBD treatment) proved to be stable and effective in the DSS-induced UC mouse model [61]. These NPs were prepared by dissolving 50 mg of LNT in 20 mL of deionized water. Additionally, 5 mg of budesonide was dissolved in 2 mL acetone. Budesonide solution was mixed with LNT solution and stirred at 300 rpm at 25 °C for 12 h to eliminate all acetone. It was found that these NPs significantly attenuated inflammation by inhibiting the TLR4/MyD88/NF-κB signaling pathway. Moreover, lentinan-based NPs with budesonide inhibited colon shortening and disease progression.

In a study by Liu et al. [62], lentinan was conjugated with selenic acid and used as a Se ion carrier. To prepare the conjugate, the authors dissolved 1.0 g of lentinan in 0.5% nitric acid (100 mL). Next, 0.8 g of sodium selenite and 1.44 g of barium chloride were added. The mixture was stirred, neutralized, dialyzed and concentrated to obtain powder form. It has been demonstrated that Se-lentinan inhibited tumor progression by regulating epithelial–mesenchymal transition. Moreover, Se-lentinan reduced the invasiveness of human colorectal adenocarcinoma cells (HCT-8). It decreased cancer cell viability, induced G0/G1 phase arrest and reduced HCT-8 cell migration and invasion. In addition, in vivo studies found that the formula inhibited tumor growth in HCT-8 colon cancer-bearing mice, prolonged overall survival time and downregulated the expression of epithelial–mesenchymal transition (EMT) markers. The EMT is a reprogramming process associated with a more aggressive CRC phenotype [63].

Ng and Yap [64] investigated the antitumor effects of orally administered lentinan against various human colon carcinoma cell lines (LoVo, SW48, SW480, SW620, SW403, SW1116) in mice. It was revealed that lentinan (3 mg per mouse per day for 7 days) significantly reduced the size of tumors.

Lentinan has also been evaluated in a model of colon cancer in BDIX rats [65]. Colonic epithelial cells from colon carcinoma (K12/PROb) were inoculated into rats. All animals exhibited peritoneal carcinomatoses. Rats received intraperitoneal injections of lentinan (2, 10 or 50 mg/kg) for 6 or 30 weeks. Lentinan-treated animals had reduced carcinomatoses when compared to controls; however, the difference was not statistically significant. Later, rats received a cumulative dosage of l0 mg lentinan/kg administered in doses of 10, 3 or 2 mg/kg. The three doses were effective in reducing the growth of carcinomatoses and inhibiting the production of ascites when administered daily.

It has been reported that inducing calcium-sensing receptors (CaSRs) increases the sensitivity of human colon carcinoma cells to anticancer drugs [66]. Therefore, in a study by Wang et al. [67], lentinan was evaluated as a CaSR modulator in human colon carcinoma cell lines: CBS, Moser, Fet and SW480. Lentinan suppressed invasion of the colon carcinoma cells and increased the cytotoxicity of chemotherapy combination that included folinic acid, fluorouracil and irinotecan. It was found that lentinan downregulated the expression of survivin (apoptosis inhibitor) and thymidylate synthase (precursor for DNA biosynthesis and target for CRC chemotherapy with 5-fluorouracil). In contrast, lentinan increased the complex formation of E-cadherin and beta-catenin (implicated in lower cancer progression). The authors concluded that lentinan effectively promotes chemosensitivity in colon carcinoma cells and could be used as a supplement for CRC therapy.

In a study conducted by Suga and Takehana [68], the effectiveness of lentinan in combination with S-1 (prodrug of 5-FU—tegafur, gimeracil and oteracil potassium) was evaluated. Lentinan (0.5 mg/mL) was intravenously or intraperitoneally administered in combination with S-1 to C26 tumor-bearing mice. It was found that S-1 + lentinan increased the number of apoptotic bodies in the colon on day 1; however, their number decreased significantly by days 2 and 5. It was associated with the impact of this drug on the increased number of CD11b^+^TIM-4^+^ cells essential for apoptotic body clearance.

In contrast, Mushiake and colleagues [69] demonstrated that chemoimmunotherapy using S-1 combined with lentinan prolonged the survival period of C26 tumor-bearing mice but not in immunodeficient athymic mice. The number of infiltrating CD86^+^ DCs cells into C26 mice was higher in mice treated with S-1 + lentinan, and these cells more potently activated T cell proliferation. In addition, cytotoxic T lymphocytes (CTLs) in S-1 + lentinan-treated mice were significantly more active. These findings strongly suggest that the anticancer effects of lentinan are associated with its immunomodulatory properties, in particular on DCs.

The great majority of the studies cited above have shown a beneficial effects of lentinan and other bioactive compound from *L. edodes* in IBD or CRC. However, Mitamura et al. [70] found that lentinan (10 µg/mouse administered three times intraperitoneally) in the azoxymethane (AOM) and DSS-treated group non-significantly tended to increase the colorectal dysplasia and splenic weight.

In the latest study, Zhang et al. [71] analyzed lentinan’s effect on colon microbiota and inflammatory parameters in mice with 5-FU-induced intestinal mucositis. Lentinan was administered orally in a dose of 5 mg/kg or 50 mg/kg. It was elucidated that it reduced weight loss (in a concentration of 50 mg/kg), increased food intake (in both concentrations) and relieved diarrhea (in both concentrations). Moreover, lentinan treatment ameliorated histopathological pathological changes in the colon, upregulated mucin and tight-junction proteins (ZO-1, Claudin-1) expression and increased the number of goblet cells. In addition, lentinan downregulated the production of pro-inflammatory cytokines, including TNF-α, IL-1 and IL-6, and upregulated IL-10 production.

Liu et al. [72] isolated lentinan from *L. edodes* fruiting bodies via alkali solvent extraction by using a 5% sodium hydroxide and 0.05% sodium borohydride and degraded it into three fractions by ultrasonication. Next, the group demonstrated that all three fractions (at concentrations of 25, 50, 100, 200 and 400 µg/mL) inhibited colon cancer cells (HT-29 and SW480) viability by enhancing cell apoptosis. Moreover, these fractions administered orally increased the body weight and colon length and inhibited immune cell infiltration in mice with AOM/DSS-induced colitis. In addition, lentinan fractions reduced the production of IL-1β, IL-6 and TNF-α in mice serum. Furthermore, all fractions regulated the gut microbiota (increased the percentage of *Bacteroides* and *Muribaculum* and decreased the fraction of harmful *Proteobacteria* and *Helicobacter*).

In another study, Mao and colleagues [73] analyzed whether lentinan supplementation could improve gut barrier dysfunction induced by rotavirus in a weaned piglet model. It was found that lentinan administered orally (84 mg/kg for 19 days) reduced diarrhea, improved the morphology of jejunal mucosa, restored their antioxidant capacity and significantly enhanced the height of the villi in a weaned piglet model. Additionally, lentinan supplementation increased the populations of *Lactobacillus, Bifidobacterium* and total bacteria in the cecal digesta of pigs.

In addition to AHCC and lentinan, other compounds from *L. edodes* have also been studied in the context of intestinal diseases. Alagbaoso and Mizuno [74] determined the impact of crude polysaccharide extract from *L. edodes* (named LeA) on inflammatory cytokines in DSS-induced colitis in mice. Crude polysaccharides were extracted from pulverized *L. edodes* fruiting body by distilled water at 100 °C for 6 h and with absolute ethanol. Later, LeA was separated into two fractions by column chromatographic separation: LeAP1 and LeAP2. LeAP1, a carbohydrate-rich active fraction (structure not presented), was the primary focus of this study. It was found that LeAP1 administered orally (300 µg/mouse) prevented body weight loss, downregulated TNF-α, IL-6, IL-8, IL-1β, IFN-γ and c-c motif chemokine ligand 2 (CCL-2) expression in the colon of mice. Additionally, suppression of pMLKL expression, a necroptosis executor in mice colonic tissue, was further detected by Western blot.

Another extract from *L. edodes* with immunomodulatory and anticancer properties is LEM [75]. LEM is a dried powder of a hot water extract of the mycelia of *L. edodes* before germination, which was cultured in a medium composed of bagasse and rice bran. LEM contains polysaccharides, proteins, nucleic acids, trace minerals, water-soluble lignin and other components. The most active components of LEM are two glycogen-like polysaccharide fractions (Mw from 50,000 to 100,000 Da). LEM’s antitumor effects have been evaluated in mice models of colon carcinoma by Tanaka et al. [76]. Colon carcinoma C26 cells were inoculated into the subserosal space of the cecum of mice. Mice were fed with LEM extract at a concentration of 1 or 2% (*w*/*w*) for 14 days. It was revealed that the growth of the C26 tumor cells was significantly suppressed in mice fed with a 2% LEM. Moreover, the extract increased the number of C26-reactive T cells in the cecum, upregulated IFN-γ levels and downregulated TGF-β production by T cells. Administration of LEM resulted in decreased levels of IL-6 and TGF-β in mice serum. These results strongly suggest that LEM can restore antitumor T cell response and can be used as an adjuvant in colon cancer immunotherapy.

*L. edodes* mycelia solid culture extract (MSCE) contains several bioactive molecules, such as sugars, proteins and polyphenolic compounds (syringic acid, vanillic acid, lignin and lignin-decomposition products) [77]. Matsuhisa et al. [78] analyzed the effect of MSCE and lignosulfonic acid on intestinal epithelial barrier function in the human colorectal adenocarcinoma cell line Caco-2. It was found that both MSCE (500 and 1000 µg/mL) and lignosulfonic acid (100 and 500 ng/mL) enhanced epithelial barrier function in Caco-2 cell monolayers by suppressing expression level of claudin-2 (a protein that forms high conductive cation pores in tight junctions). Additionally, it was revealed that lignosulfonic acid attenuated TNF-α/IFN-γ-induced NF-κB activation and downregulated IL-1β and IL-6 expression in Caco-2 cells.

Numerous studies suggested that probiotics and fiber have beneficial effects in CD and UC therapy [79,80,81]. Therefore, the objective of the study conducted by Xue et al. [82] was to analyze the symbiotic effects of soluble dietary fiber (SDF) obtained from *L. edodes* and *Lactobacillus plantarum* (LP90) on DSS-induced UC model of mice and its effects on intestinal barrier permeability, Th17/Treg cytokines balance and gut microbiota. SDF and LP were administered orally for 8 days (250 mg/kg/day and −1 × 10^9^ CFU/kg/day, respectively). Orally administered SDF + LP decreased disease activity, prevented weight loss, increased colon length and reduced diarrhea. In addition, the treatment upregulated IL-10 and immunoglobulin (IgA) and reduced TNF-α, IFN-γ and IL-17A production. Moreover, butyric acid synthesis and diversity of intestinal microbiota were increased.

Improvement of intestine function by modulation of the gut microbiome has also been demonstrated in a study by Li and colleagues [83]. The authors isolated a glucan-rich polysaccharide (named LePS40) from *L. edodes*, which consisted of a β-(1→3)-D-glucan backbone with (1→6)-linked glucose side chains. Next, the digestive and fermentative outcomes of LePS40 in human gastrointestinal tract environments and fecal microbiota were analyzed in vitro. It was found that LePS40 is resistant to digestive enzymes and gastric acid; however, it is easily fermented. The polysaccharide increased butyric and propionic acid production and decreased the amount of bacteria associated with IBD and CRC, including *Faecalibacterium, Anaerostipes, Ruminococcus* and *Prevotella*.

All of the results summarized in Table 1 suggest that various bioactive compounds from *L. edodes* ameliorate the pathological symptoms of intestinal disorders, regulate the content of gut microbiota, protect intestinal barrier function, decrease pro-inflammatory response and reduce oxidative stress. However, future studies are needed to elucidate potential mechanisms associated with observed effects.

### 3.2. Studies Performed in Humans

Numerous bioactive compounds from functional foods, including glucans, were suggested to influence the intestinal microbiome and immune system [84,85]. Therefore, the aim of a controlled, randomized, double-blind clinical study was to analyze the immunomodulatory and microbiota-altering effect of *L. edodes* extract in 52 patients with untreated mild hypercholesterolemia [86]. Study participants consumed 10.4 g/day of β-D-glucan-enriched (BGE) mixture (containing 3.5 g of fungal β-D-glucans) isolated from fruiting bodies of *L. edodes* or a placebo incorporated in three different soups. BGE is a mixture of two extracts containing water-soluble α- and β-D-glucans, fucomannogalactans, as well as insoluble β-D-glucans and chitins. After eight weeks, no significant differences were observed in the IL-1β, IL-6 and TNF-α production between the analyzed groups. However, the extract administration resulted in the modulation of colonic microbiota. In the BGE-treated group, an increase in microbial diversity was observed. This is a significant finding, as reduced microbial diversity in IBD has been correlated with disease severity [87].

The first randomized efficacy study of lentinan on patients with advanced-stage gastric cancer and CRC was conducted in 1986 by Wakui et al. [88]. A total of 166 patients, including 115 with gastric cancer and 51 with CRC, were enrolled. It was found that lentinan, in combination with mitomycin C and 5-fluorouracyl, prolonged the survival time of patients with advanced stages of gastric cancer and CRC.

In another randomized, placebo-controlled trial, Satake et al. [89] evaluated the efficacy of *L. edodes* mycelia extract (LEM) in oxaliplatin-induced peripheral neuropathy (OIPN) in 44 CRC patients. Participants were divided into three groups: 15 received a placebo, 15 low dose (300 mg twice a day) and 14 high dose (900 mg twice a day) of LEM for 12 weeks, starting 3 months after postoperative oxaliplatin-based chemotherapy. No significant difference in the visual analog scale (VAS) numbness was detected in the low dose vs. placebo groups. However, a statistically significant reduction in VAS numbness and pain was observed in the high-dose group. Moreover, patients receiving the high-dose treatment reported an improvement in walking problems. The potential mechanisms responsible for the observed effects need further investigation in a larger study population.

Similarly, Okuno and Uno [90] investigated the influence of LEM on adverse events from chemotherapy and its immunomodulatory properties in seven colorectal and one gastric cancer patients. During 8 weeks, each participant took two courses of chemotherapy (5-fluorouracil), irinotecan, uracil and tegafur, levofolinate, mitomycin or taxol. LEM was orally ingested during the second course at a dose of 1800 mg/day continuously for 4 weeks. Adverse events up to grade 2 were observed in the first course, however, not in the second course (LEM ingestion). Therefore, it has been suggested that LEM can be used as an oral adjuvant for CRC treatment. In addition, a slight increase in IFN-γ production by T CD4^+^, T CD8^+^ and NK/NKT CD56^+^ cells was observed during LEM treatment.

Most of the results summarized in Table 2 strongly suggest that *L. edodes* bioactive substances can be used as functional natural agents, which may favor the prevention or treatment of IBD and CRC. However, further mechanisms for preventing intestinal disorders by these agents must be revealed in future studies.

## 4. Discussion

This is the first review article that provides a critical summary of the current state of knowledge about the role of various bioactive compounds from *L. edodes* on GIT and outlines future perspectives for their use in IBD and CRC therapy.

IBD significantly increases the risk of developing CRC [20]. The exact mechanism behind this association is not fully understood; however, chronic intestinal inflammation, oxidative stress and dysbiotic gut microbiota-derived metabolites, which induce DNA damage and dysplasia, have been described as the most important risk factors [21,22,23,24]. Taking the above into consideration, improving intestinal barrier function and maintaining a more diverse and balanced gut microbiome are crucial for preventing and treating chronic inflammatory conditions of the intestine.

Despite significant research progress in IBD and CRC therapy, some patients are resistant to standard drugs or experience severe side effects that prevent them from continuing treatment. Nowadays, there is a growing need to find effective, available and affordable therapeutic options, including natural products. Medicinal mushrooms, such as *L. edodes*, are an attractive source of various compounds with therapeutical potential [33,34].

Previous studies on the relationship between *L. edodes* and intestinal inflammation or carcinogenesis have explored multiple aspects, including the impact on immune cells, oxidative stress, composition of gut microbiota, as well as angiogenesis and apoptosis of cancer cells.

The antioxidant potential of lentinan (β-(1,3)-D-glucan isolated from the fruiting bodies of *L. edodes*) and AHCC (standardized extract of *L. edodes* mycelia containing α- and β-glucans, amino acids, lipids and minerals) has been demonstrated in normal colon cells, as well as in colon cancer cells and in mice models. Both compounds decreased MPO levels and downregulated NO production [42,43,44,60]. It is worth emphasizing that antioxidants can modulate different signal transduction pathways and gene expression to inhibit oxidative stress. Previous studies demonstrated that lentinan enhanced cellular tolerance to oxidative damage and had protective effects on damaged cells by increasing glutathione activity, downregulation of malondialdehyde (MDA) formation and enhancing superoxide dismutase (SOD) activity [91]. The antioxidant mechanism of AHCC has not been fully elucidated; however, recently, it was suggested that this extract regulates the TLR2-stress-activated protein kinase/Jun NH2-terminal kinase (SAPK/JNK) pathway [92].

Lentinan, AHCC, as well as LeAP (crude polysaccharide extract from fruiting bodies), LEM (dried hot water extract from mycelia) and MSCE (mycelia solid culture extract containing proteins, sugars and polyphenols), had potent anti-inflammatory effects, both in studies on cell lines and in mice. Although it was not specified which component of the extracts shows such activity, it is believed that the α- and β-glucans, through complex interactions with multiple receptors present on immune cells (dectin-1, CR3, CD38, LaCer, TLRs and scavenger), inhibited production of pro-inflammatory cytokines (including IL-1β, IL-6, TNF-α) by downregulation of NFκB activation [42,43,44,45,46,48,49,51,52,53,55,61,69,72,74,76,76,78,82].

In addition, several studies revealed that lentinan and LePS40, β-(1→3)-D-glucan with (1→6)-linked glucose side chains restored gut microbiome heterogeneity [52,53,72,73,82,83] and improved colon tissue morphology in animal models of IBD [45,46,61,67,71,72,73,78,82]. The observed effects are most likely related to the ability of these compounds to increase the levels of propionic and butyric acids, increasing the expression of tight-junction proteins and their anti-inflammatory properties.

The anticancer effects of lentinan and other glucans isolated from *L. edodes* fruiting bodies and mycelium have been demonstrated in vitro and in mice inoculated with various colon cancer cells. These compounds enhanced apoptosis of cancer cells and inhibited tumor angiogenesis via increasing caspase 3-, 8- and 9-, and enhancing tumor infiltration of IFN-γ-producing immune cells [42,43,49,51,55,59,62,64,65,67,68,70,72].

Numerous reports suggested that dietary fiber has a beneficial effect on intestinal inflammation [79,80,81]. Therefore, the effects of soluble dietary fiber (SDF) from *L. edodes* were also studied, and it was found that this compound administered with probiotics improved intestine function and decreased disease activity by modulation of the gut microbiome by increasing the butyric acid synthesis and by reducing pro-inflammatory cytokines production in UC model of mice [82].

Improvement of intestine function by modulation of the gut microbiome has also been demonstrated in a study by Li and colleagues [83]. LePS40, similarly to SDF, increased butyric and propionic acid production and decreased the amount of bacteria associated with IBD and CRC.

Data on the therapeutical effects of *L. edodes* compounds in humans are limited; however, Morales et al. [86], in a randomized, controlled, double-blind clinical trial, demonstrated that consumption of β-D-glucan-enriched (BGE) mixture isolated from *L. edodes* increased microbial diversity in humans, which was most likely indirectly related to the increased intake of fiber. Moreover, it was found that lentinan (administered intravenously) in combination with standard chemotherapy prolonged the survival time of gastric cancer and CRC patients [87]. Another compound tested in combination with chemotherapeutic agents was LEM. LEM administered orally reduced the adverse effects of these drugs and increased IFN-γ production by T and NK cells [88,89]. Therefore, it was suggested that these compounds can be used as oral adjuvants for cancer treatment.

## 5. Conclusions and Future Perspectives

In the last decades, the immunomodulatory and anticancer effects of various polysaccharides from *L. edodes* have been described [93]. As summarized in the present review, numerous studies conducted on animal models, cell lines, as well as in humans suggested that bioactive compounds isolated from *L. edodes* fruiting bodies or mycelium can be used as adjuvants for IBD or CRC treatment. However, there are some limitations of these studies. One of them is the lack of explanation of the mechanisms responsible for the observed effects. Moreover, in many analyses, different strains of *L. edodes* have been used. In addition, divergent methods of cultivation, various isolation and purification techniques and different routes of administration are key factors that could have influenced the obtained results. It is worth emphasizing that still only a few active compounds from *L. edodes* have been purified and characterized, including lentinan and LePS40. In multiple in vitro and in vivo studies, extracts from fruiting bodies or mycelium were used (for example, AHCC and MSCE), which makes it impossible to assess which component is responsible for the observed biological activity.

Research conducted in humans has utilized small sample sizes; thus, for validation of the use of *L. edodes*-derived compounds in the treatment of intestinal diseases, larger sample sizes are needed.

Therefore, future studies must be focused on understanding the pathways and mechanisms associated with the observed effects. Moreover, both randomized trials and long-term follow-up studies are needed to show the efficacy of treatment of IBD and CRC with *L. edodes*-derived bioactive compounds.

## Figures and Tables

**Table 1:** The effects of *L. edodes*-derived bioactive compounds on colon cancer cell lines and intestinal barrier and gastrointestinal diseases in animal models.

*L. edodes* Compound	Results	References
AHCC ^1^ 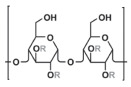 The active compound of AHCC: α-1,4-glucan (R:H or CH_3_CO-)	AHCC dose-dependently increased Nrf2 and NF-κB activity in human colon cancer cell lines (HCT116 and DLD-1). AHCC dose-dependently upregulated mRNA expression of HO-1 and NQO and reduced IL-6 mRNA expression. AHCC administered in the diet of Apc mutant Min mice decreased the number of intestinal polyps and lowered IL-6 production.	[42,43]
	AHCC reduced LPS-induced gut inflammation in rats (downregulated IL-1, IL-6 and TNF-α concentration). AHCC decreased MPO levels in the gut, downregulated NO production, reduced lymphocyte infiltration and gut edema.	[44]
Lentinan 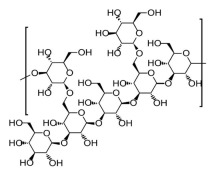 1-3;1-6-β-D-glucan	Lentinan (intraperitoneally injected in a dose of 5 mg/kg, 10 mg/kg or 20 mg/kg) in a dose-dependent manner reduced weight loss, disease activity index and colon shortening in mice with DSS-induced colitis. Lentinan increased glutathione activity, downregulated malondialdehyde and TNF-α, IL-6 and IL-1β levels in colon. Lentinan, in combination with probiotics, increased colon cell proliferation, reduced apoptosis and blocked the NF-κB pathway.	[45]
	Lentinan downregulated IL-8 mRNA expression and NF-κB activation in Caco-2 cells. Lentinan decreased the number of TNFR1 on Caco-2 cells. Orally administered lentinan (100 µg/day) reduced weight loss, prevented shortening of the colon length and downregulated IFN-γ, IL-1β and TNFR1 mRNA intestinal expression in mice.	[46]
	Orally administered lentinan (100 µg/day) induced recruitment of Th and Treg cells from an ileum into a colon and non-significantly downregulated IFN-γ, IL-1β, IL-4, IL-10 and TNF-α expression in mice with DSS-induced colitis.	[48]
	Lentinan induced cell apoptosis of HT-29 human colon cancer cells in vitro and in mice by caspase-3, -8 and -9 activation. Lentinan upregulated cytochrome c and the Bax/Bcl-2 ratio, reduced NF-κB activation, ROS levels and TNF-α production in vitro and in vivo. Lentinan (1 and 5 mg/kg administered intraperitoneally) reduced tumor weight in immunodeficient NOD/SCID mice. Lentinan upregulated expression of autophagy-related proteins (LC3-II and Beclin-1) in HT-29 cells.	[49,51]
	Orally administered lentinan (200 mg/kg/day) reversed the dysbiosis induced by ampicillin, neomycin sulfate, metronidazole and vancomycin in mice. Lentinan increased the levels of propionic and butyric acids, improved colon tissue morphology, increased the expression of tight-junction proteins such as ZO-1 and Occludin in mice and downregulated pro-inflammatory cytokines production via the NF-κB signaling pathway.	[52]
	Lentinan effectively restored the gut microbiome heterogeneity in a mouse model of IBD and CAC. Lentinan decreased the disease activity index and colon tissue damage and reduced the number of tumors, colon cancer markers, immune cell infiltration and expression of IL-13.	[53]
	Lentinan treatment (1.0 mg/kg) reduced tumor angiogenesis and tumor growth in mice inoculated with CT26 colorectal cells via upregulation of IFN-γ production.	[55]
	Lentinan-functionalized SeNPs (LNT-SeNPs, 2–64 μM) inhibited the proliferation of HCT-116, HT-29, Caco-2, SW620 and CT26 colon cancer cells. The strongest effect was observed for the HCT-116 cells. LNT-SeNPs increased apoptosis of HCT-116 cells and induced G0/G1 phase arrest in these cells.	[59]
	LNT-SeNPs (1–64 μM) decreased CT26 mouse colon carcinoma cell viability and induced excessive ROS production in a dose-dependent manner. LNT-SeNPs induced apoptosis and G0/G1 phase arrest in CT26 cells. LNT-SeNPs, in combination with TNF-α, inhibited tumor growth in CT26 tumor-bearing mice.	[60]
	Lentinan-based oral NPs loaded with budesonide attenuated inflammation by inhibiting the TLR4/MyD88/NF-κB signaling pathway in DSS-induced UC mouse model. Lentinan-based NPs with budesonide inhibited colon shortening and disease progression.	[61]
	Lentinan conjugated with selenic acid reduced the invasiveness of human colorectal adenocarcinoma cells (HCT-8). It decreased cancer cell viability, induced G0/G1 phase arrest and reduced HCT-8 cell migration and invasion. Lentinan conjugated with selenic acid inhibited tumor growth in HCT-8 colon cancer-bearing mice, prolonged overall survival time and downregulated the expression of EMT markers.	[62]
	Orally administered lentinan (3 mg per mouse per day for 7 days) reduced tumor size in LoVo-, SW48-, SW480-, SW620-, SW403- and SW1116-tumor-bearing mice.	[64]
	Lentinan (2, 10 or 50 mg/kg intraperitoneally injected) reduced carcinomatoses in rats with K12/PROb colon carcinoma.	[65]
	Lentinan suppressed invasion of colon carcinoma CBS, Moser, Fet and SW480 cells and increased the cytotoxicity of a chemotherapy combination that included folinic acid, FU and irinotecan in vitro. Lentinan downregulated expression of survivin and thymidylate synthase and increased complex formation of E-cadherin and beta-catenin.	[67]
	Intravenously or intraperitoneally administered lentinan (0.5 mg/mL) in combination with chemotherapy increased apoptosis of cancer cells in C26 tumor-bearing mice.	[68]
	Lentinan combined with chemoimmunotherapy increased the number of infiltrating CD86^+^ DCs cells in C26 mice, and these cells more potently activated T cell proliferation. Lentinan combined with chemoimmunotherapy increased activity of CTLs.	[69]
	Lentinan (10 µg/mouse administered three times intraperitoneally) non-significantly tended to increase the colorectal dysplasia and splenic weight in the AOM- and DSS-treated mice.	[70]
	Orally administered lentinan (5 mg/kg or 50 mg/kg) reduced weight loss (in concentration of 50 mg/kg), increased food intake (in both concentrations) and relieved diarrhea (in both concentrations) in mice with 5-FU-induced intestinal mucositis. Lentinan ameliorated histopathological pathological changes in colon, upregulated mucin and tight-junction proteins (ZO-1, Claudin-1) expression and increased the number of goblet cells. Lentinan downregulated production of pro-inflammatory cytokines, including TNF-α, IL-1 and IL-6, and upregulated IL-10 production.	[71]
	Lentinan administered orally (84 mg/kg for 19 days) reduced diarrhea, improved morphology of jejunal mucosa, restored their antioxidant capacity and significantly enhanced the height of the villi in a weaned piglet model. Lentinan supplementation increased the populations of Lactobacillus, Bifidobacterium and total bacteria in the cecal digesta of pigs.	[73]
β-glucan from *L. edodes* degraded into three fractions	All three fractions (at concentrations of 25, 50, 100, 200 and 400 µg/mL) inhibited colon cancer cells (HT-29 and SW480) viability by enhancing cell apoptosis. All three fractions increased the body weight and colon length, inhibited immune cell infiltration in mice with AOM/DSS-induced colitis and reduced the production of IL-1β, IL-6 and TNF-α in mice. All three fractions increased microbial diversity in mice.	[72]
LeAP1 and LeAP2 (crude polysaccharide fractions extracted from *L. edodes*)	LeAP1 administered orally (300 µg/mouse) prevented body weight loss and downregulated TNF-α, IL-6, IL-8, IL-1β, IFN-γ and CCL-2 expression in the colon of mice. LeAP1 suppressed pMLKL expression in mice colonic tissue.	[74]
LEM (dried powder hot water extract from *L. edodes* mycelia)	LEM administered orally (2% (*w*/*w*) for 14 days) suppressed the growth of colon carcinoma C26 cells in mice and increased the number of C26-reactive T cells in the cecum. LEM upregulated IFN-γ levels and downregulated TGF-β and IL-6.	[76]
MSCE (*L. edodes* mycelia solid culture extract)	MSCE (500 and 1000 µg/mL), in combination with lignosulfonic acid (100 and 500 ng/mL), enhanced epithelial barrier function in Caco-2 cells by suppressing expression level of claudin-2. MSCE + lignosulfonic acid attenuated TNF-α/IFN-γ-induced NF-κB activation and downregulated IL-1β and IL-6 expression in Caco-2 cells.	[78]
Soluble dietary fiber (SDF) from *L. edodes*	SDF and Lactobacillus plantarum (LP90) administered orally for 8 days (250 mg/kg/day and −1 × 10^9^ CFU/kg/day, respectively) decreased disease activity, prevented weight loss, increased colon length and reduced diarrhea in DSS-induced UC model of mice. SDF and Lactobacillus plantarum upregulated IL-10 and IgA levels and reduced TNF-α, IFN-γ and IL-17A production. SDF and Lactobacillus plantarum upregulated butyric acid synthesis and diversity of intestinal microbiota.	[82]
LePS40 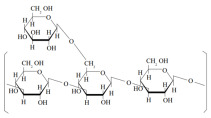 β-1,3-D-glucan with 1,6-linked glucose side chains	LePS40 increased butyric and propionic acid production and decreased the amount of bacteria associated with IBD and CRC, including Faecalibacterium, Anaerostipes, Ruminococcus and Prevotella.	[83]

^1^ AHCC, Active Hexose Correlated Compound; CAC, colitis-associated cancer; CRC, colorectal cancer; CTLs, cytotoxic T lymphocytes; DSS, dextran sulfate sodium; EMT, epithelial–mesenchymal transition; FU, fluorouracil; HO, heme oxygenase; IBD, inflammatory bowel disease; IFN, interferon; IL, interleukin; LPS, lipopolysaccharide; MPO, myeloperoxidase; MSCE, mycelia solid culture extract; NQO1, NAD(P)H:quinone oxidoreductase; NF-E2, nuclear factor-erythroid 2Nrf2, p45-related factor; NK, natural killer; NKT, natural killer; ROS, reactive oxygen species; SeNPs, selenonanoparticles; SCID, severe combined immunodeficiency; SDF, soluble dietary fiber; TNF, tumor necrosis factor; TGF, transforming growth factor; UC, ulcerative colitis; ZO, zonula occludens.

**Table 2:** The effects of *L. edodes*-derived bioactive compounds on intestinal barrier and gastrointestinal diseases in humans.

Treatment	Number of Participants and Condition	Results	Reference
β-D-glucan-enriched (BGE) mixture (containing 3.5 g of β-D-glucans) isolated from *L. edodes* consumed 10.4 g/day for 8 weeks	52 patients with untreated mild hypercholesterolemia and dysbiosis	BGE administration increased microbial diversity	[86]
Lentinan (2 mg/week administered intravenously) in combination with mitomycin and 5-fluorouracil	115 gastric cancer and 51 CRC ^1^ patients	Lentinan, in combination with chemotherapy, prolonged the survival time of gastric cancer and CRC patients	[87]
*L. edodes* mycelia extract (LEM) administered orally 300 mg twice a day (low dose) or 900 mg twice a day (high dose) for 12 weeks	29 CRC patients with oxaliplatin-induced peripheral neuropathy	High-dose LEM administration reduced VAS numbness and pain and improved walking problems	[88]
LEM orally ingested during the second course of chemotherapy (5-fluorouracil), irinotecan, uracil and tegafur, levofolinate, mitomycin or taxol) at a dose of 1800 mg/day for 4 weeks	7 CRC patients and one gastric cancer patient	LEM ingestion reduced adverse effects of chemotherapy and increased IFN-γ production by T CD4^+^, T CD8^+^ and NK/NKT CD56^+^ cells	[89]

^1^ CRC, colorectal cancer; IFN, interferon; NK, natural killer; NKT, natural killer; VAS, visual analog scale.

## Abbreviations

The following abbreviations are used in this manuscript:

AHCC	Active Hexose Correlated Compound
CAC	Colitis-associated cancer
CaSR	Calcium-sensing receptor
CD	Crohn’s disease
CTLA-4	Cytotoxic T-lymphocyte-associated protein 4
CRC	Colorectal cancer
DCs	Dendritic cells
DSS	Dextran sulfate sodium
EGFR	Epidermal growth factor receptor
FOXP3	Forkhead box P3
GIT	Gastrointestinal tract
IBD	Inflammatory bowel disease
ILCs	Innate lymphoid cells
IFN	Interferon
IL	Interleukin
LEM	Lentinula edodes mycelia extract
LNT	Lentinan
MPO	Myeloperoxidase
NFκB	Nuclear factor κB
NK	Natural killer
NO	Nitric oxide
ROS	Reactive oxygen species
SCID	Severe combined immunodeficiency
SeNPs	Selenonanoparticles
Tc	T cytotoxic cells
Th	T helper cells
Tm	T memory cells
TNF	Tumor necrosis factor
TGF	Transforming growth factor
UC	Ulcerative colitis
WT	Wild type
ZO	Zonula occludens
